# Age‐related and species‐specific methylation changes in the protein‐coding marmoset sperm epigenome

**DOI:** 10.1111/acel.14200

**Published:** 2024-05-16

**Authors:** Marcus Dittrich, Laura Bernhardt, Christopher A. Penfold, Thorsten E. Boroviak, Charis Drummer, Rüdiger Behr, Tobias Müller, Thomas Haaf

**Affiliations:** ^1^ Institute of Human Genetics Julius Maximilians University Würzburg Germany; ^2^ Department of Bioinformatics Julius Maximilians University Würzburg Germany; ^3^ Department of Physiology, Development and Neuroscience University of Cambridge Cambridge UK; ^4^ Centre for Trophoblast Research University of Cambridge Cambridge UK; ^5^ Wellcome Trust – Medical Research Council Stem Cell Institute, Jeffrey Cheah Biomedical Centre University of Cambridge Cambridge UK; ^6^ Platform Degenerative Diseases German Primate Center‐Leibniz Institute for Primate Research Göttingen Germany; ^7^ DZHK (German Centre for Cardiovascular Research) Göttingen Germany

**Keywords:** glycosphingolipid biosynthesis pathway, male germ cells, marmoset, non‐human primate, paternal age effect, sperm methylome, transcription start site

## Abstract

The sperm epigenome is thought to affect the developmental programming of the resulting embryo, influencing health and disease in later life. Age‐related methylation changes in the sperm of old fathers may mediate the increased risks for reproductive and offspring medical problems. The impact of paternal age on sperm methylation has been extensively studied in humans and, to a lesser extent, in rodents and cattle. Here, we performed a comparative analysis of paternal age effects on protein‐coding genes in the human and marmoset sperm methylomes. The marmoset has gained growing importance as a non‐human primate model of aging and age‐related diseases. Using reduced representation bisulfite sequencing, we identified age‐related differentially methylated transcription start site (ageTSS) regions in 204 marmoset and 27 human genes. The direction of methylation changes was the opposite, increasing with age in marmosets and decreasing in humans. None of the identified ageTSS was differentially methylated in both species. Although the average methylation levels of all TSS regions were highly correlated between marmosets and humans, with the majority of TSS being hypomethylated in sperm, more than 300 protein‐coding genes were endowed with species‐specifically (hypo)methylated TSS. Several genes of the glycosphingolipid (GSL) biosynthesis pathway, which plays a role in embryonic stem cell differentiation and regulation of development, were hypomethylated (<5%) in human and fully methylated (>95%) in marmoset sperm. The expression levels and patterns of defined sets of GSL genes differed considerably between human and marmoset pre‐implantation embryo stages and blastocyst tissues, respectively.

## Graphical abstract

Using reduced representation bisulfite sequencing, we identified age‐related differentially methylated transcription start site regions in 204 marmoset and 27 human genes. All identified ageTSS were species‐specific, suggesting flexible evolutionary epigenetic adaptations. Although overall, the TSS methylation levels of orthologous genes were highly correlated in both species, there were also notable between‐species differences, that is, in sperm methylation and postzygotic expression of genes for glycosphingolipid biosynthesis.

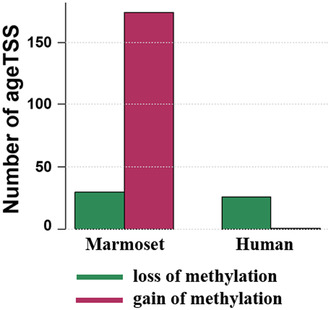

**Abbreviations**
ageTSS: age‐related differentially methylated TSS
CJA: *Callithrix jacchus*
GSL: Glycosphingolipid
HSA: *Homo sapiens*
MDS: Multi‐Dimensional Scaling
RRBS: Reduced Representation Bisulfite Sequencing
TSS: Transcription Start Site

## 1 INTRODUCTION

Advanced paternal age is associated with increased risks for reproductive and offspring medical problems. This appears to be due to declining sperm quality rather than quantity. The chances of aging men to achieve a pregnancy are also reduced (Hassan & Killick, 2003). The offspring's risk for some rare monogenic disorders due to de novo genetic mutations (Crow, 2000) as well as for complex neurodevelopmental disorders and impaired neurocognitive outcomes (De Kluiver et al., 2017) increases with paternal age. Accumulating evidence suggests age‐related changes in the sperm epigenome as one underlying mechanism. In the continuously dividing male germline, the number of spermatogonial cell divisions increases from 35 times at puberty to >800 times at the age of 50 years (Crow, 2000). During each replication cycle, not only the DNA sequence itself but also its epigenetic marks must be correctly copied to the daughter cells. Since the error rate of this copying process is at least one order of magnitude higher for epigenetic than for genetic information (Bennett‐Baker et al., 2003), the spermatozoa from older males are endowed with many more epigenetic than DNA sequence changes.

Several conceptually related genome‐wide studies have reported age‐associated methylation changes in the human sperm epigenome (Denomme et al., 2020; Jenkins et al., 2014; Laurentino et al., 2020; Oluwayiose et al., 2021). Using reduced representation bisulfite sequencing (RRBS), we have identified >1000 genes whose sperm methylation level decreases or less frequently increases with the donor's age (Bernhardt et al., 2023). Altogether, 2355 genes have been reported with age‐associated methylation changes in eight published sperm methylation screens. However, only 241 (10%) of the 2355 genes were found in at least two independent studies, which makes them primary candidates for paternal age effects on the sperm epigenome. These 241 genes showed significant functional enrichments in biological processes associated with the development of the nervous system and in cellular components associated with synapses and neurons (Bernhardt et al., 2023). This supports the hypothesis that the aging sperm methylome affects offspring behavior and neurodevelopment (Denomme et al., 2020; Jenkins et al., 2014; Laurentino et al., 2020; Oluwayiose et al., 2021).

Because of the long generation time and limited access to the critical tissues in humans, mouse models have been used to study shared epigenetic signatures between aged fathers and resulting offspring and their impact on brain gene expression, behavior, and age‐related pathologies (Milekic et al., 2015; Yoshizaki et al., 2021). However, considering the huge differences in developmental gene expression (Boroviak et al., 2018), reproduction, and aging physiology/pathology between humans and rodents, it is difficult to extrapolate findings in the mouse model to humans. Preliminary evidence from methylation studies of orthologous genes in humans, bovine, and mice suggests that paternal age effects on the sperm epigenome are largely species‐specific and under epigenomic evolution (Prell et al., 2022).

Our closest relatives are non‐human primates. The common marmoset, *Callithrix jacchus* (CJA), is a small New World monkey that represents an excellent model for studying reproduction, aging, neurodevelopment, and infectious disease (Tardif et al., 2011). In contrast to larger, more difficult to handle, less reproductive, and more expensive primate models, such as the rhesus macaque, marmosets have a small adult body size (250–500 g) and compressed lifespan (10–15 years in captivity). Like humans, they live in families. Sexual maturity of males is reached by 19 months (Chandolia et al., 2006), and the first offspring is usually produced by 2–3 years. The prime adult age ranges from 2 to 8 years, and old age from 8 to 15 years (Abbott et al., 2003). Although marmosets and humans diverged >40 million years ago, their karyotypes and genome sequence characteristics are surprisingly similar (Yang et al., 2021). To find out whether or not age‐related methylation changes in the human sperm epigenome have been conserved in non‐human primates, we have performed a genome‐wide RRBS analysis of marmoset sperm samples from 20 months to 12.5‐year‐old donors.

## 2 RESULTS

### 2.1 Ordination

**FIGURE 1:**
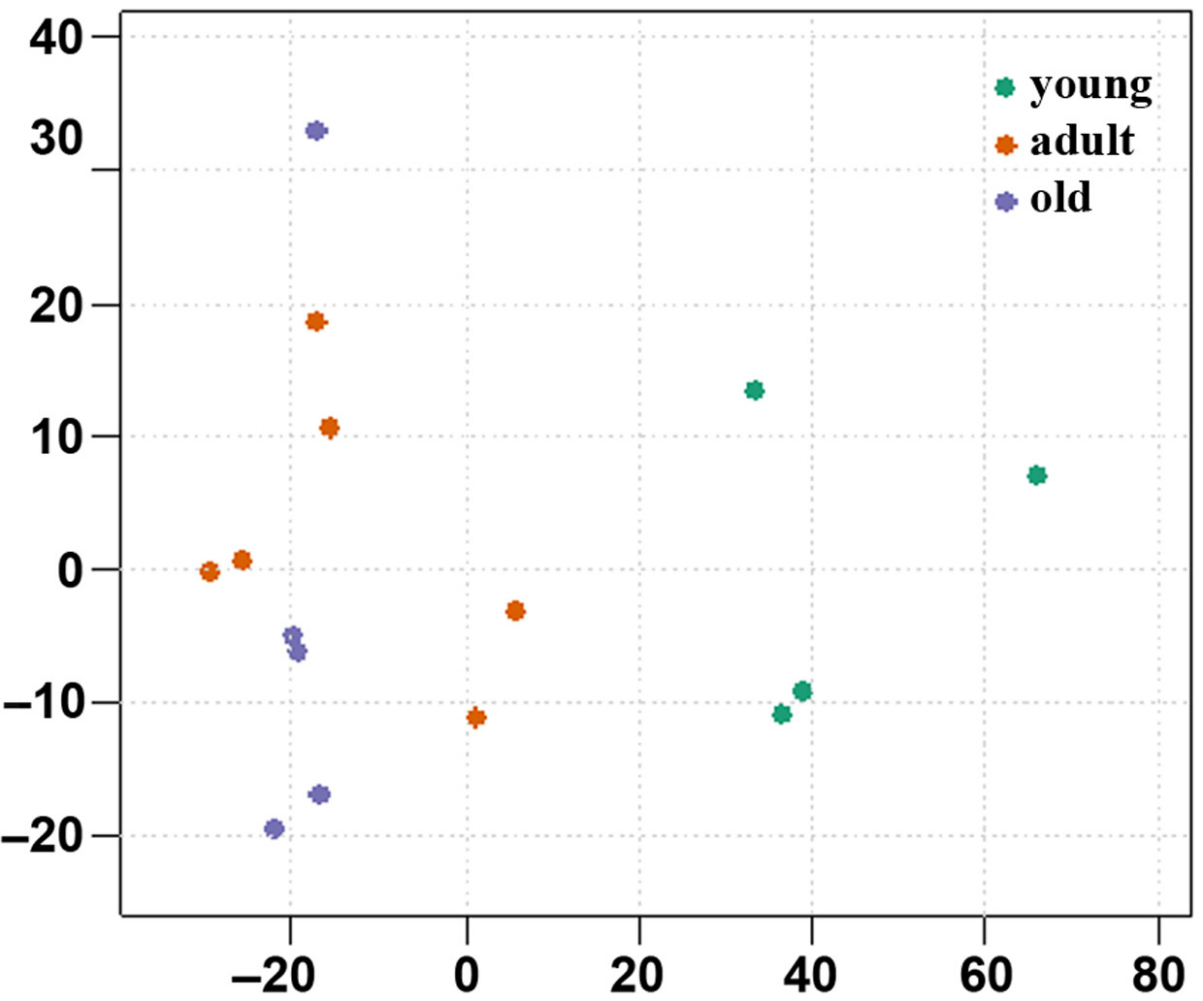
Multi‐dimensional scaling (MDS) analysis of 15 *Callithrix* sperm samples was based on the M‐values of the 1000 top variant TSS regions. Green dots represent samples from four animals younger than 2 years (sexual maturity group), red dots from six animals between 2 and 8 years (prime adults), and mauve dots from five animals between 8 and 12 years (old age group). MDS revealed age as the main effect on the first axis and showed a clear separation of the youngest group samples. There was no separation between adult and old individuals.

RRBS was performed on 15 CJA sperm samples. Four samples from donors between 20 and 24 months were classified as “Young,” 6 from donors between 2 and 8 years as “Prime Adult,” and five from donors between 8 and 13 years as “Old Age.” Histology of testes of all animals showed complete spermatogenesis in all tubules, and epididymides were completely filled with spermatozoa (Figure S1). To obtain an overview of the data and detect potential global effects, we performed a Multi‐Dimensional Scaling (MDS) analysis of all samples using the 1000 top variant transcription start site (TSS) regions (Figure 1). The MDS analysis revealed age as a major global effect and separated the young individuals on the first axis. However, there was no obvious separation between adult and old individuals.

### 2.2 Comparison of TSS methylation patterns in marmoset and human sperm

To compare age‐associated methylation changes between CJA and humans, we furthermore re‐analyzed a previously published RRBS data set from 73 human sperm samples (Bernhardt et al., 2023). First, we analyzed the methylation of regions around the TSS of all protein‐coding genes in each species individually and subsequently performed a comparative analysis of the genes with a unique orthologue in each species (as detailed in Section 4.3). To identify age‐related differentially methylated regions in protein‐coding genes, methylation was aggregated over the TSS region of each gene. These analyses yielded significant age‐related differentially methylated TSS regions (ageTSS) for 204 (1.5%) of 13,914 covered genes in CJA (Table S1) and for 27 (0.2%) of 13,718 genes in the human data set (Table S2). Notably, the methylation differences were in largely opposite directions (Figure S2). In marmosets, the vast majority (174 of 204; 85%) of sperm ageTSS gained methylation with age, whereas in humans, the methylation of essentially all (26 of 27; 96%) ageTSS decreased with age. The enrichment analysis of the ageTSS in marmosets yielded no significant pathways. For a genome‐wide analysis of humans, we refer to our previous study (Bernhardt et al., 2023). To compare age‐related methylation patterns between species, we focused on 9905 genes with a unique orthologue in each species (for more details, see Section 4.3). However, none of these conserved genes, including 85 ageTSS in marmosets and 18 in humans, showed a significant age effect in both species. A scatter plot of differential methylation values (model‐based age effects on the M‐value scale) of humans and marmosets showed only a very weak correlation of the age effects between species (Figure S3).

### 2.3 Bimodal distribution of TSS methylation levels in human and marmoset sperm and its effects on embryonal gene expression

**FIGURE 2:**
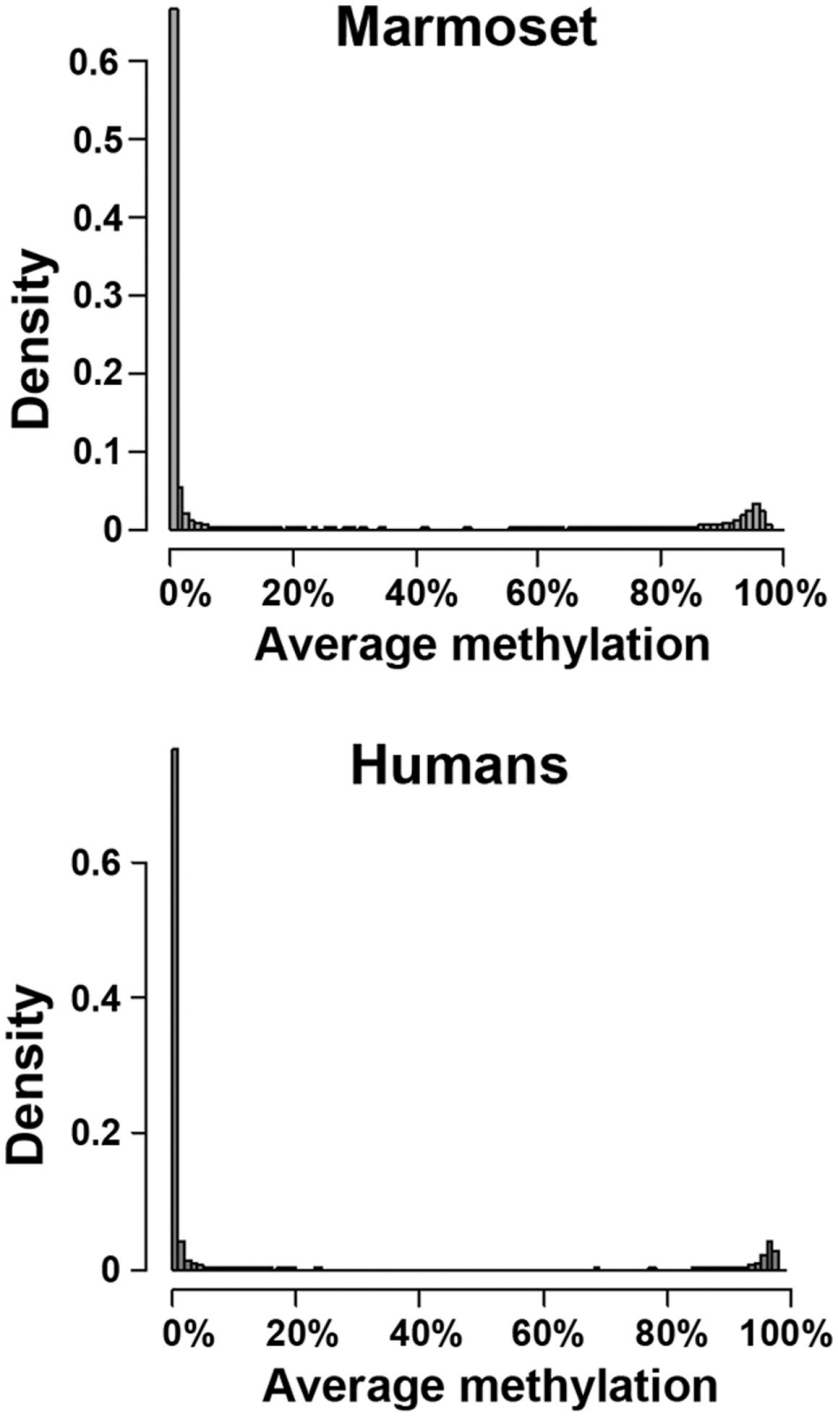
The histograms show the average methylation levels of TSS regions in marmosets (top) and humans (bottom). Most genes are almost completely unmethylated or, to a lesser extent, almost fully methylated in both species.

**FIGURE 3:**
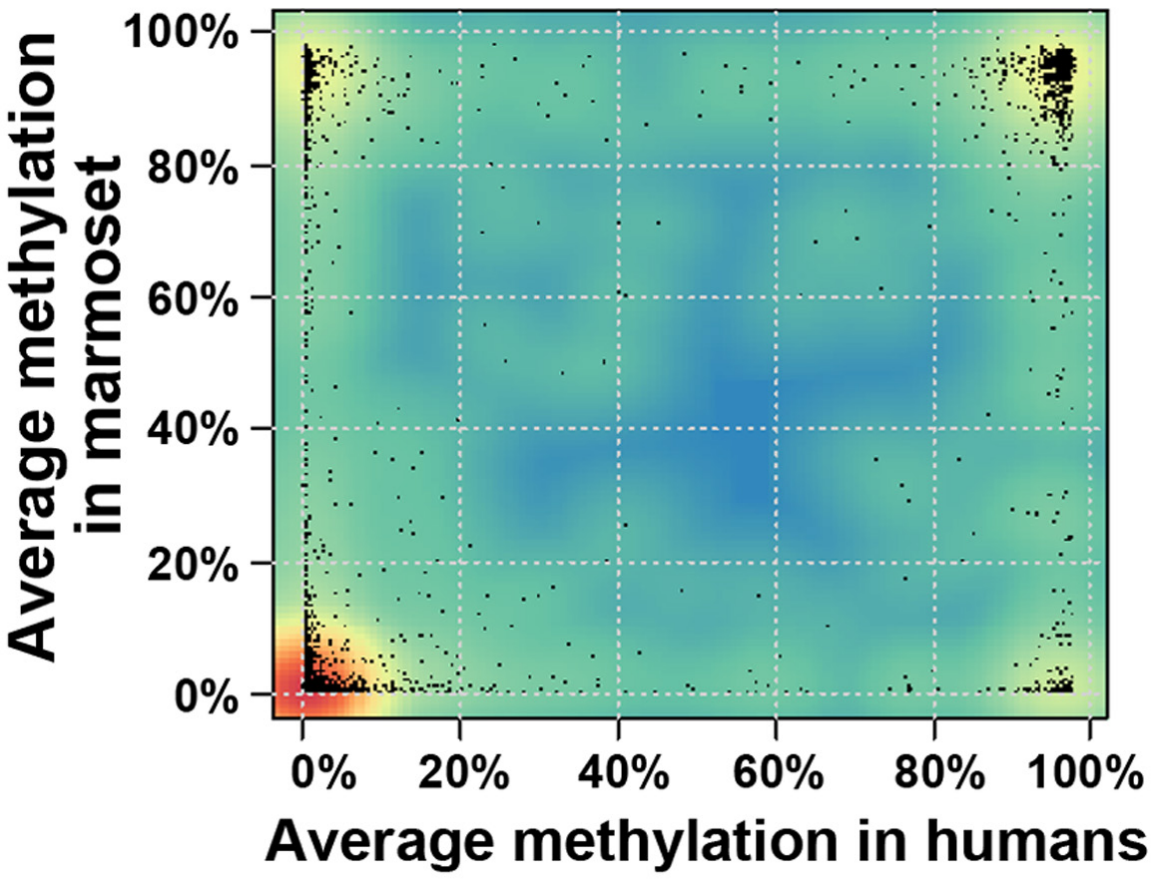
The density plot compares the TSS methylation of all analyzed orthologous genes between marmosets and humans. Red colors denote regions of high density, blue colors low density. The methylation values of individual genes are indicated by black dots. Most genes are unmethylated in both species and cluster in the left bottom corner, or fully methylated in both species, clustering in the right top corner. Overall, the methylation values are significantly correlated between the species (Pearson's correlation rho = 0.61, *p* < 2.2E−16). Nevertheless, there are 132 orthologous genes whose TSS regions are unmethylated in marmosets and fully methylated in humans (right bottom corner) and 197 genes with unmethylated TSS in humans and methylated TSS in marmosets (left top corner).

Overall, the distribution of DNA methylation levels of TSS regions of all protein‐coding genes was highly similar in both species: most regions were either completely unmethylated (average methylation over entire regions <5%) or, to a lesser extent, fully methylated (> 95%) (Figure 2). In marmosets, 10,576 out of 13,914 (76%) analyzed TSS regions were unmethylated, and 880 (6.3%) were fully methylated in sperm. Similarly, in humans, 11,468 out of 13,718 (84%) analyzed TSS regions were hypomethylated, and 1217 (9%) were hypermethylated in sperm. Subsequently, we performed a comparative analysis of the 9905 orthologous TSS regions, which were covered in both species. This yielded a highly significant correlation of the average methylation levels between both species (Pearson's correlation rho = 0.611, *p* < 2.2E−16) indicating, that on a global level, the patterns of gene regulation have been conserved between marmoset and human sperm (Figure 3).

**FIGURE 4:**
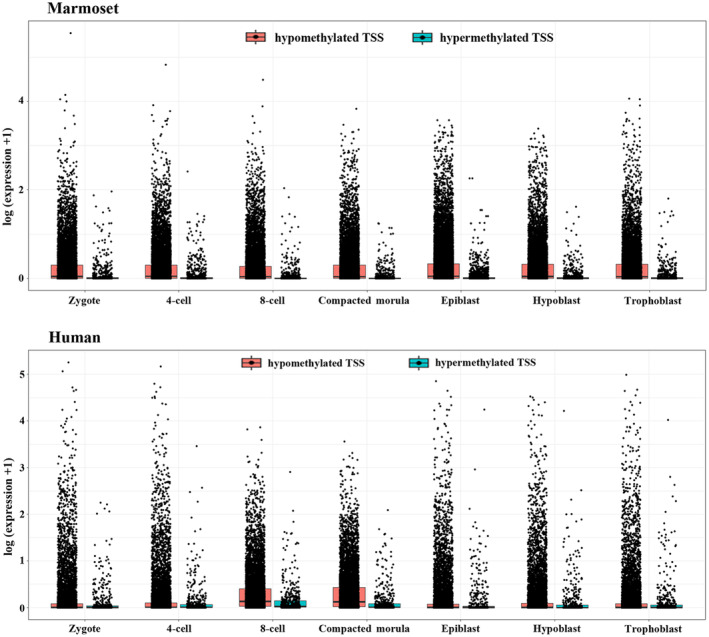
Relationship between gene expression (in early embryos and blastocyst tissues) and sperm methylation levels, as visualized using ggplot. Genes are first stratified into highly methylated (>95%) and lowly methylated (<5%) genes. Expression levels are plotted for various tissues for the highly and lowly methylated genes as box plots. Expression of highly methylated genes is significantly lower (Wilcoxon test) than that of their lowly methylated counterparts for all tissues from the zygote to the blastocyst stage in both marmosets and humans. In humans, *p* values are 0.00034 for the zygote, 0.015 for four‐cell embryo, 2.2E−6 for eight‐cell embryo and compacted morula, 0.014 for epiblast, 7.6E−5 for hypoblast, and 0.0029 for trophoblast. In marmosets, all *p* values are 2.2E−16, which is the precision limit of the test in R.

To test whether the TSS methylation levels in sperm affect embryonal gene expression, we re‐analyzed marmoset and human single‐cell transcriptomes, using DESeq2 (Love et al., 2014). To this end, we used published data sets from preimplantation embryos (zygote, four‐cell stage, eight‐cell stage, compacted morula), and blastocyst lineages (epiblast, hypoblast, and trophectoderm) of marmosets (Bergmann et al., 2022; Stirparo et al., 2018) and humans (Blakeley et al., 2015; Petropoulos et al., 2016; Yan et al., 2013). Because of the strong bimodal distribution of methylation levels, with 76–84% of the TSS regions being fully demethylated (<5%) and 6–9% fully methylated (>95%), but not many with moderate methylation levels, we have visualized the expression levels for the hypomethylated and the hypermethylated groups side‐by‐side as box plots (Figure 4). We noted statistically significant (Wilcoxon test) lower expression in highly methylated genes than their lowly methylated counterparts for all tissues from the zygote to blastocyst stage in both human and marmoset.

### 2.4 Species‐specifically (hypo)methylated TSS regions

Interestingly, 132 (1.6%) CJA genes with an unmethylated TSS region have a human orthologue with fully methylated TSS (Table S3; Figure 3), and vice versa, 197 (2.2%) human genes with unmethylated TSS have a fully methylated marmoset orthologue (Table S4; Figure 3). To investigate the functions of these genes with species‐specific TSS methylation, we performed pathway enrichment analyses within the marmoset genome. For the marmoset‐specifically unmethylated genes, no enriched pathways could be detected. For the set of marmoset‐specifically methylated genes, KEGG pathway 00603, Glycosphingolipid (GSL) biosynthesis—globo and isoglobo series, was significantly enriched (adjusted *p* = 0.024). Three genes in this pathway, *B3GALT5*, *ST3GAL1*, and *ST3GAL2*, were endowed with TSS, showing <5% methylation in human and >95% methylation in marmoset sperm.

### 2.5 Differential expression of GSL biosynthesis in human and marmoset early embryos

**FIGURE 5:**
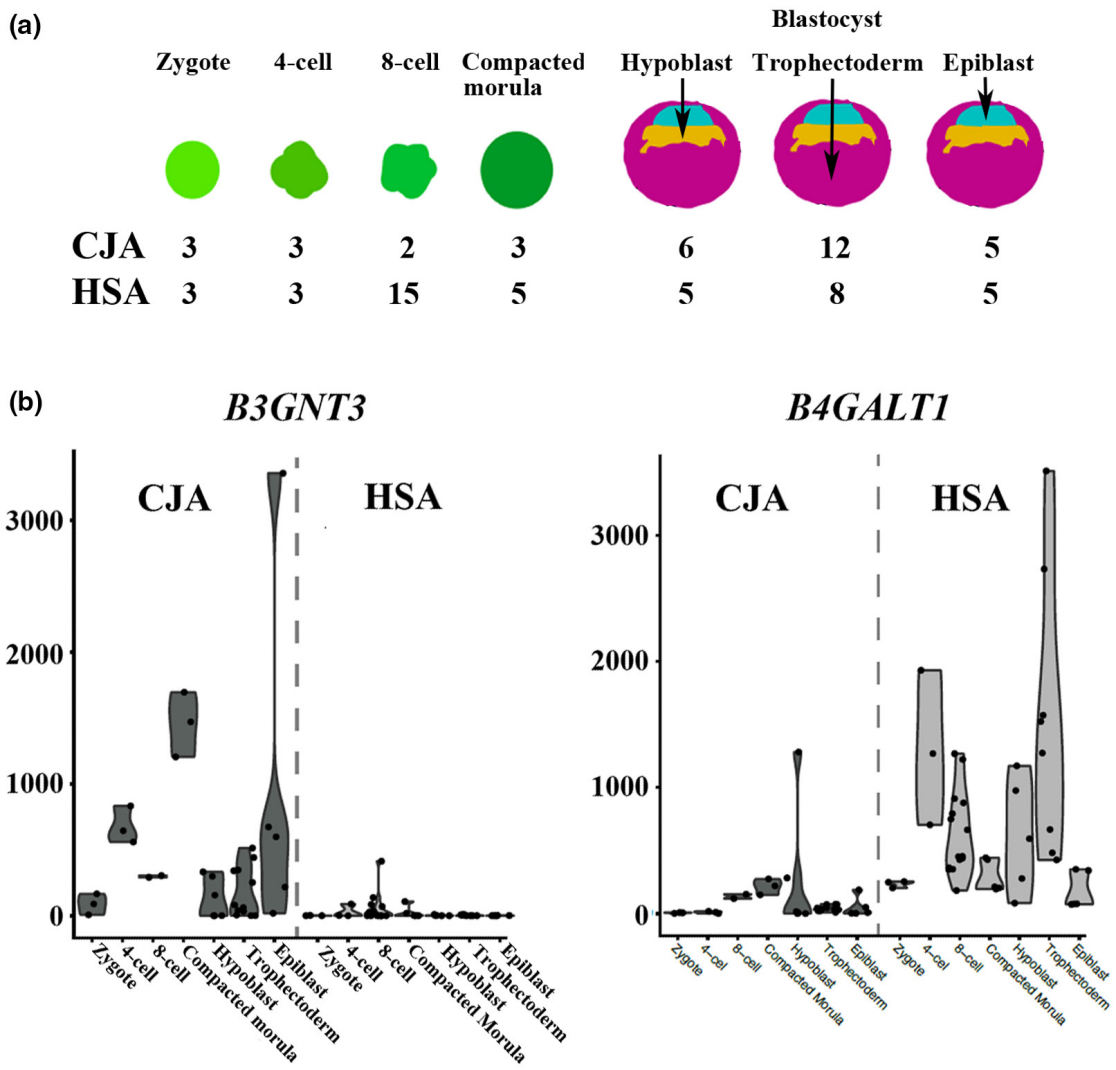
(a) Schematic drawing and number of the analyzed embryo stages and blastocyst tissues. (b) Violin plots of embryo or tissue level expression of *B3GNT3* and *B4GALT1* as graphic examples for a gene that is upregulated in marmosets (CJA) and in humans (*Homo sapiens*, HSA), respectively.

**FIGURE 6:**
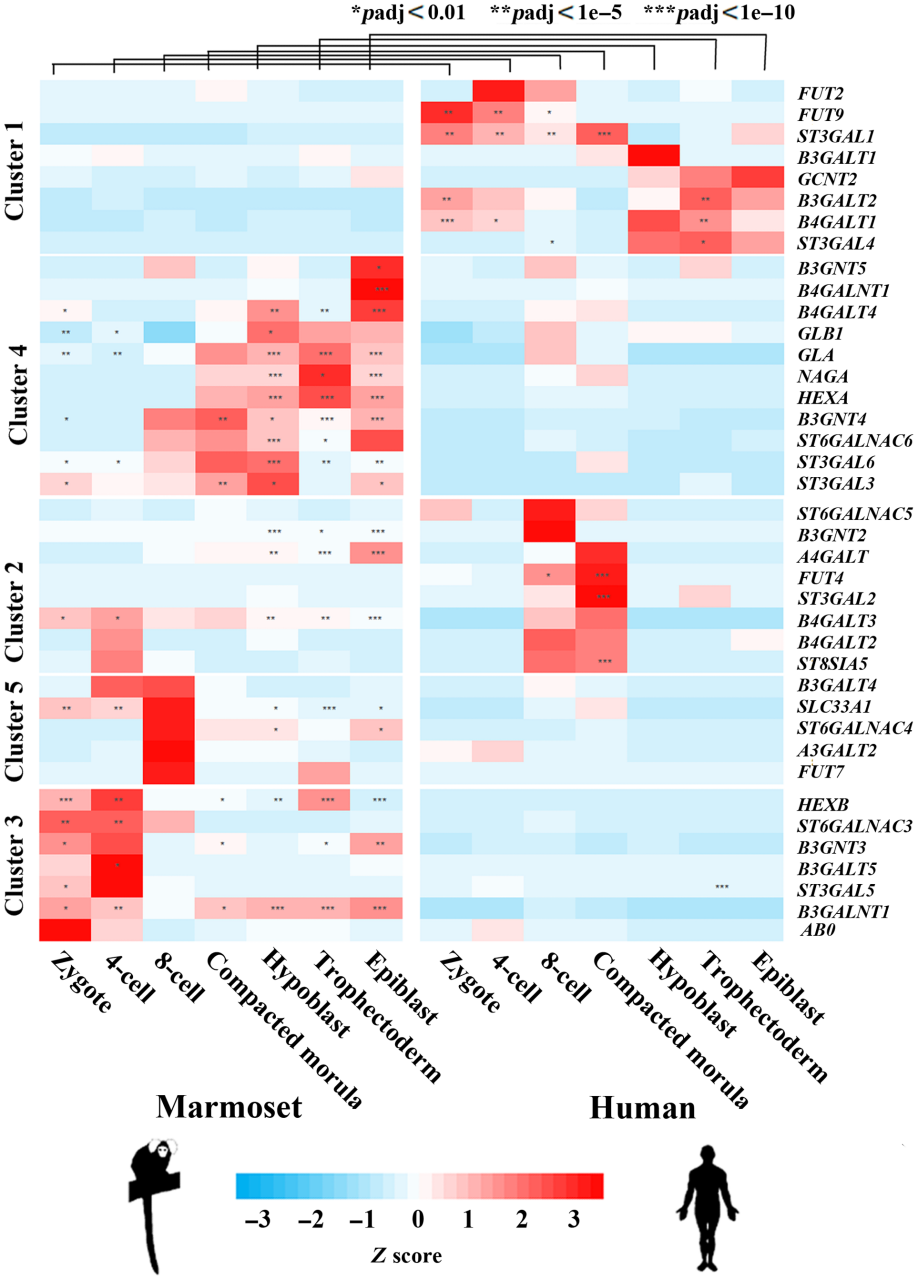
Heatmap of relative expression levels of genes relating to GSL biosynthesis (KEGG pathway 00603) in human (right side) and marmoset (left) preimplantation embryos. Analyzed stages are from left to right zygote, four‐cell stage, eight‐cell stage, compacted morula, and three blastocyst tissues, hypoblast, trophectoderm, and epiblast. Preimplantation development shows stage‐specific differential expression between marmoset and human (adj. *p < 0.01; **p < 1E2−5, ***p < 1E−10).

To investigate the GSL biosynthesis pathway further, we looked at the expression levels in the above‐mentioned single‐cell RNA‐seq data sets, using DSeq2. The number of individual embryos for each species and each sample is highlighted in Figure 5a. Relative expression levels for each stage were visualized as a heatmap for humans and marmosets (Figure 6) and clustered using a hierarchical clustering approach (for more details, see Section 4.4). Differential expression between marmosets and humans for each stage (or tissue) was used to highlight statistically significant (* adjusted *p* < 0.01; ** adj. *p* < 1E−5, *** adj. *p* < 1E−10) upregulated genes.

We identified a number of modules that demonstrate specific divergence between marmosets and humans. There were five main clusters. Cluster 1 demonstrated upregulation in humans at the zygotic and four‐cell stages, including *FUT9* and *ST3GAL1*, or in the trophectoderm, including *B3GALT2*, *B4GALT1*, and *ST3GAL4*. Cluster 2 showed upregulation of human genes over the compacted eight‐cell and morula, including *FUT4*, *ST3GAL2*, and *ST8GSIA5*. The other three modules were upregulated to some degree in marmosets, with cluster 3 showing elevated expression in the zygotic and four‐cell stages (*HEXB*, *ST6GALNAC3*, *B3GALT5*, *ST3GAL5*, and *B3GALNT1*) and cluster 4 showing higher expression from the compacted morula stage and in the blastocyst (*B3GNT5*, *B4GALNT1*, *B4GALT4*, *GLA*, *NAGA*, *HEXA*, *B3GNT4*, *ST6GALNAC6*, *ST3GAL6*, and *ST3GAL3*). As a graphic example, individual counts for the marmoset upregulated gene, *B3GNT3*, and the human upregulated gene, *B4GALT1*, are shown in Figure 5b.

## 3 DISCUSSION

In this study, we performed a comparative sperm methylation analysis of TSS regions in humans and a New World monkey. Arguably, genes represent the most important functional units of the genome, and their regulatory regions are around TSS. Orthologous genes that are conserved at the protein level usually also have conserved functions in different species. To investigate the patterns of regulation of conserved genes, we focused our analyses on protein‐coding genes with unique orthologues in both species.

### 3.1 Conserved and species‐specific TSS methylation patterns in sperm

Consistent with other mammalian species (Fang et al., 2019; Moharrek et al., 2022; Qu et al., 2018), sperm methylation in marmoset displayed a bimodal distribution with the majority of promoter regions being hypomethylated (76%) or hypermethylated (6%). Overall, the TSS methylation levels of orthologous genes were highly correlated in marmosets and humans, indicating a common epigenetic signal in their sperm methylomes.

Although most sperm methylation patterns have been conserved between CJA and humans, there are also notable between‐species differences. It is generally assumed that germ‐cell methylation influences the transcriptional activation of genes in embryo development (Smith et al., 2012). In this light, species‐specifically (hypo)methylated sperm TSS are thought to be associated with genes under epigenomic evolution, contributing to species‐specific phenotypic traits and adaptation to different environments. It has been reported that genes with human‐specifically hypomethylated sperm promoters are associated with the development and function of the nervous system, cattle‐specific promoters with lipid storage and metabolism, and mouse‐specific promoters with the perception of smell (Fang et al., 2019; Moharrek et al., 2022).

### 3.2 The interrelation of sperm chromatin structure and epigenetic changes

Nucleosomes in mammalian sperm have been associated with developmentally important genes with hypomethylated promoters, instructing embryonic development and transmitting acquired epigenetic changes/phenotypes to the next generation (Gaspa‐Toneu & Peters, 2023; Hammoud et al., 2009). Most of the sperm genome is packaged by protamines into a highly condensed, almost crystalline structure. The minor fraction retaining a more open nucleohistone structure is assumed to be more susceptible to accumulating epigenetic changes during aging and environmental exposures (Ashapkin et al., 2023). Indeed, it has been reported that genes with age‐associated methylation changes are enriched in regions, retaining nucleosomes in sperm (Bernhardt et al., 2023; Denomme et al., 2020). In this light, nucleosomal packaging may be a mechanism to stabilize advantageous epigenetic changes by making them accessible to the enzymatic machinery for modifying DNA methylation during spermatogenesis.

Similar to genetic mutations, age‐associated epigenetic changes may be subject to selective processes. The vast majority of random epigenetic changes, which occur during the replication of histone‐packaged DNA in spermatogonial stem cells, may be neutral or even disadvantageous and, therefore, not (positively) selected by epigenomic evolution. However, if a particular epigenetic change is advantageous for embryonal development or environmental adaptations, it may become fixed, that is, by a more open chromatin structure facilitating the establishment or maintenance of an acquired epigenetic mark during spermiogenesis. The mechanisms driving epigenome evolution are poorly understood. According to the concept of antagonistic pleiotropy, epigenetic changes that are advantageous, that is, by allowing environmental adaptations at the species level, may be selected even if they are associated with disadvantageous traits, that is, an increased risk for neurodevelopmental disorders.

### 3.3 Species differences in the GSL pathway in human and marmoset embryos

In this study, we found that several genes of the GSL biosynthesis pathway were methylated in marmoset and unmethylated in human sperm. Re‐analysis (using DESeq2) of single‐cell transcriptomes from human and marmoset embryos and blastocyst tissues allowed us to look at the KEGG pathway 00603 more generally, defining gene clusters which are specifically upregulated in human and others which are upregulated in marmoset embryogenesis. Interestingly both human‐specifically upregulated genes, that is, *ST3GAL1* (cluster 1) and *ST3GAL2* (cluster 2), and marmoset upregulated genes, that is, *B3GALT5* (cluster 3), were endowed with human‐specifically hypomethylated sperm ageTSS. DNA methylation is essential for embryonic development and differentiation (Suzuki & Bird, 2008). In general, promoter methylation is associated with gene silencing by preventing the binding of transcription factors. However, in some cases, in particular, during tumor development hypomethylation can also promote gene silencing (Guo et al., 2023). In this light, species‐specifically (hypo)methylated sperm ageTSS may contribute to the observed expression differences of the GSL pathway, involving both upregulation and downregulation of specific gene sets in marmoset and human embryogenesis, respectively.

GSLs are a highly heterogenous group of plasma membrane lipids with sugar moieties attached to a ceramide backbone, which can modulate cellular interactions and contribute to embryo development. Specific GSLs interact with specific protein domains in signaling molecules and regulate their activities. This is essential for the control of signaling in development and differentiation, modulating cellular adhesion, growth, and motility (Russo et al., 2016; Yu et al., 2016). The GSL expression patterns, reflecting stage‐specific transitions during differentiation processes of undifferentiated human embryonal cells, depend on the expression of distinctive sets of GSL synthesizing enzymes. The precursor cells for given differentiation lineages express different GSLs on their cell surface and, therefore, may respond differently to morphogenetic signals, resulting in alternative differentiation fates. During neurogenesis, for example, there is a switch in GSL synthesis from the globo‐ to lacto‐series to primarily gangliosides (Liang et al., 2010).

Knockout mouse models for particular GSL synthesizing enzymes present with specific developmental defects (D'Angelo et al., 2013). In addition, specific GSLs play pivotal roles in various age‐related human pathologies, including cancer, autoimmune, metabolic, cardiovascular, and neurodegenerative disease (Balram et al., 2022). Collectively, it is plausible to assume that the observed species differences in the methylation of genes of the GSL synthesizing pathway in the sperm epigenome affect embryonal cell differentiation. Interestingly, embryo development in CJA is much slower, compared to humans (Lázaro et al., 2023).

### 3.4 No shared signal of paternal age in the marmoset and human sperm epigenomes

The sperm epigenome is plastic and susceptible to environmental factors, in particular, paternal age (Denomme et al., 2020; Jenkins et al., 2014; Laurentino et al., 2020; Oluwayiose et al., 2021). Accumulating evidence in both humans (Atsem et al., 2016; Potabattula et al., 2018) and mouse (Milekic et al., 2015; Yoshizaki et al., 2021) suggests that sperm epigenetic alterations can be transmitted to the next generation, affecting offspring health. In this light, environmentally induced epigenetic variation in the sperm epigenome may represent a mechanism to enhance phenotypic plasticity in the offspring and to shape species‐specific adaptation to environment (Moharrek et al., 2022; Prell et al., 2022), that is, neurodevelopment in humans. The mechanisms by which sperm epigenetic alterations can be transmitted to the next generation are still largely unclear. Some non‐imprinted loci may also be resistant to postzygotic methylation reprogramming after fertilization (Lane et al., 2003) or, if the marks are completely erased, they may be re‐established.

Previously, we have studied paternal age effects on the sperm epigenome in humans, bovine, and mice. In contrast to rDNA and other repetitive DNA families, which showed an evolutionarily conserved gain of DNA methylation with age in all analyzed species (Potabattula et al., 2020), paternal age effects on single‐copy genes appeared to be species‐specific (Prell et al., 2022). In this study, we performed a genome‐wide comparison of age‐associated methylation changes in TSS regions of protein‐coding genes between marmosets and humans. Collectively, our results support the idea of species‐specific paternal age effects on the protein‐coding sperm epigenome. In *Callithrix*, most (85%) ageTSS gain methylation with age, whereas in humans, almost all (96%) ageTSS are downmethylated with age. There was no overlap between orthologous genes with significant age‐related methylation changes in marmosets and humans.

### 3.5 Saltatory aging of the marmoset sperm epigenome

The total length of spermatogenesis in the common marmoset was reported to be 37 days (Millar et al., 2000). Even if this duration is underestimated, as in the sibling species *Callithrix penicillata* it was calculated to be 69 days, it is reasonable to assume that the total length of spermatogenesis in the common marmoset does not exceed 70 days. Male common marmosets complete puberty between postnatal weeks 50 (first tubules with elongated spermatids) and 72 (all tubules show quantitatively and qualitatively normal spermatogenesis) (Chandolia et al., 2006). By postnatal week 72, the first wave of spermatogenesis has been completed. Adding an additional 70 days to this age (maximum assumed duration of spermatogenesis in the common marmoset), it can be assumed that latest by postnatal week 82 (~19 months), only sperm from normal ongoing spermatogenesis (i.e., no sperm from the first wave) are produced. The animals in our young group were all between 20 and 24 months of age and showed complete spermatogenesis in histological sections of the testes and epididymides. Thus, we assume that all semen samples analyzed in this study represent the physiology of stabilized adult spermatogenesis.

The dynamics of age‐associated methylation changes appeared to differ between marmosets and humans. In marmosets, the most dramatic changes in the sperm epigenome occurred between young males who have reached sexual maturity and adults. In MDS analysis, there was no clear separation between adults (2–8 years) and old animals (9–12 years). This “saltatory” aging of the sperm epigenome which occurs within a few months after sexual maturity, may be associated with the CJA mating and breeding system, which differs from human reproduction. Monogamous male and female marmoset pairs form stable breeding groups. Usually, only a single dominant male and female pair breed, whereas all group members aid in rearing the offspring (Abbott et al., 2003). In humans, most studies present models with linear effects of paternal age on the methylation of individual genes. However, the donor age usually ranges somewhere from 20 to 80 years. The samples analyzed here were from 25‐ to 51‐year‐old donors. There are no conclusive studies on the human sperm methylome changes of 14–18 year old donors, corresponding to the sexual maturity group in marmosets.

By using RRBS (unpublished data), we have also identified a considerable number of age‐associated methylation changes in mouse and human sperm. Similar to humans, there appeared to be a linear increase or decrease of DNA methylation at the identified ageDMRs in both species. The lifespan of marmosets lies somewhere between that of mice (28 months) and bovine (20 years). Thus, the compressed life span of marmosets and, consequently, the lower number of spermatogonial cell divisions, compared to humans, does not explain the different dynamics of sperm epigenetic aging.

### 3.6 Conclusions

Here, we report four novel findings: (1) There is a saltatory switch in the methylation of a number of genes in marmoset sperm during adolescence; the corresponding developmental ages in humans have not been analyzed so far. (2) The two primate species examined in this study show mutually exclusive sets of sperm ageTSS; none of the identified ageTSS was differentially methylated in both species. This suggests flexible evolutionary epigenetic adaptations. (3) The direction of age‐dependent methylation changes is inverse, increasing in marmosets and decreasing in humans. (4) Although overall, the TSS methylation levels of orthologous genes are highly correlated in the marmoset and human, there are also notable between‐species differences. The species‐specific differential TSS methylation of genes important for GSL biosynthesis between humans and marmosets is reflected in the different abundance of transcripts in the early embryos of the two species.

## 4 EXPERIMENTAL PROCEDURES

### 4.1 Study samples

Fifteen sperm samples from common marmosets (*Callithrix jacchus*) were obtained by penile vibrostimulation of 20 months to 12.5‐year‐old animals housed at the German Primate Center in Göttingen. Animal experiments were approved by the Niedersächsisches Landesamt für Verbraucherschutz und Lebensmittelsicherheit (no. 42502‐04‐17/2496). Ejaculation was stimulated using a FertiCare personal vibrator (Multisept ApS, Rungsted, Denmark) fitted with a 2 cm × 0.5 cm tube with rounded edges that served as an artificial vagina. Sperm collection medium consisting of HEPES‐buffered Tyrode's lactate with 0.25 mM sodium pyruvate (Sigma‐Aldrich, Darmstadt, Germany) and 0.3% wt/vol Bovine Serum Albumin (Sigma‐Aldrich), pH 7.3, 400 μL, 37°C was immediately added to the ejaculate to minimize coagulation of the seminal plasma. In the laboratory, sperm samples were washed through the 40%/80% density gradient prepared with PureSperm 100 solution (Nidacon, Mölndal, Sweden) and sperm collection medium, during 18 min centrifugation using soft mode at 300*g* and 37°C. Finally, the cell pellet was carefully placed on the bottom of a tube filled with 600 μL TALP medium and placed in the incubator at 37.5°C, 5% O_2_, and 5% CO_2_ for 40 min. Swim‐up sperm were collected, visually inspected under the microscope, and used for DNA isolation.

Seventy‐three human semen samples, most (56) of them from males with normal semen parameters, were collected at the Fertility Center Wiesbaden (for more details, see Bernhardt et al., 2023). After IVF/ICSI treatment, the left‐over swim‐up sperm fraction (excess material) was pseudonymized, and snap‐frozen at −80°C until further use. To eliminate contamination by bacteria, lymphocytes, epithelial, and other somatic cells, the swim‐up sperm samples were gently thawed and purified further by density gradients PureSperm 80 and 40 (Nidacon). The study was approved by the ethics committee at the medical faculty of the University of Würzburg (no. 117/11 and 212/15). Written informed consent was obtained from each donor.

For DNA isolation, the purified sperm cells were resuspended in 300 μL buffer (5 mL of 5 M NaCl, 5 mL of 1 M Tris–HCl; pH 8, 5 mL of 10% SDS; pH 7.2, 1 mL of 0.5 M EDTA; pH 8, 1 mL of 100% β‐mercaptoethanol, and 33 mL of H_2_O), and 100 μL (20 mg/mL; 600 mAU/mL) proteinase K (Qiagen, Hilden, Germany), and incubated for 2 hours at 56°C. Sperm DNA was isolated using the DNeasy Blood and Tissue kit (Qiagen). DNA concentration and purity were measured with a Qubit fluorometer (Thermofisher, Massachusetts, USA).

### 4.2 Reduced representation bisulfite sequencing

RRBS enriches areas of the genome with high CpG content and, therefore, reduces the costs of whole genome bisulfite sequencing by only sequencing a reduced representative subset of the genome, which still contains the majority of promoters and other important genomic features. RRBS libraries were generated from 15 marmoset sperm samples using the commercially available Ovation RRBS Methyl‐Seq System 1–16 (Tecan, Männedorf, Switzerland). Briefly, genomic DNA was digested by the methylation‐sensitive restriction enzyme MspI, followed by adapter ligation and a final repair step. Then, bisulfite conversion was performed using the EpiTect Fast DNA Bisulfite Kit (Qiagen, Hilden, Germany) for low DNA concentrations. After PCR amplification and bead purification of the final library, DNA concentration was measured with the HS‐DNA Kit and Qubit fluorometer. Fragment length distribution was assessed by running the samples on the Bioanalyzer 2100 (Agilent, Waldbronn, Germany) using a High Sensitivity DNA Chip. Finally, 2 × 76 paired‐end sequencing of 15 samples in parallel was performed on the NextSeq500 platform using the NextSeq 500/550 High Output v2 Kit (150 cycles) (Illumina, California, USA).

### 4.3 Bioinformatic analysis of marmoset and human RRBS data sets

After sequencing of the libraries, quality control was performed with FastQC (version v0.11.3) and QC reports have been summarized with multiQC (Figure S4). Adapter trimming was performed using TrimGalore (version 0.4.0), followed by subsequent trimming of diversity adapters according to the manufacturer's protocol and scripts (trimRRBSdiversityAdaptCustomers.py, version 1.11). The human RRBS data set was obtained from a previous study (Bernhardt et al., 2023). Human and the marmoset data were processed correspondingly. The marmoset reads were mapped to the *Callithrix* genome (mCalJac1.pat.X, INSDC Assembly GCA_011100555.1) obtained from the Ensembl database using Bismark (version 22.3) (Krueger & Andrews, 2011), with the bowtie2 option activated. Methylation levels of CpG sites were calculated based on the number of reads supporting the methylated and unmethylated state using the methylation extractor script of the Bismark suite. Only CpG sites covered in all samples have been used further.

For comparative analysis, the TSS regions were defined based on the gene catalog from the Ensembl database. Therefore, the position of TSS was obtained for all protein‐coding transcripts of each gene. They were extended to include the flanking 1000 bp upstream and downstream sequence. As many genes may comprise multiple transcripts and transcription start sites of different genes may be in close vicinity to each other, transcripts with start sites closer than 2 kb to a transcription start site of another gene were discarded to obtain a unique assignment of measured CpG sites to the genes. Subsequently, for each gene, the TSS region with the largest number of CpGs measured in the data set was selected, and the methylation level was calculated as the weighted average of the methylation overall CpGs in the region. Only regions with a coverage of five or more in all samples and a non‐zero variability of beta values across the samples were included in downstream analyses, yielding a total of 13,718 regions for humans and 13,914 for marmosets. For differential methylation analysis (to detect the ageTSS) the M‐transformed methylation levels of these regions were used and regressed against the nominal age of the species using age as a numeric predictor. *P* values were obtained with a moderated t‐test as implemented in the linear modeling framework Limma. MDS analysis has been performed on the M‐values with the routines implemented in the Minfi package (Aryee et al., 2014), using default settings. Therefore, the 1000 top variant TSS were identified based on the variance of the M‐transformed methylation levels across the entire cohort independent of age and thus in an unbiased manner. Multiple testing correction was performed using the Benjamini‐Hochberg method. All statistical analyses were performed using R (version 3.6.3) including packages from the Bioconductor project.

For all genes in the CJA genome, the human orthologue was identified based on the orthology information in the Ensembl Compara Database (Oxford 2016:2016:baw053. doi: 10.1093/database/baw053). Only orthologies flagged as high confidence and genes with unique orthologues (denoted as ortholog_one2one mapping in Ensembl Compara) were used for comparative analyses. Pathway enrichment analyses have been performed with the gprofiler webserver (https://biit.cs.ut.ee/gprofiler/gost), setting the organism to CJA and using the set of genes covered in the marmoset for ageTSS and the set of conserved genes for species‐specifically methylated TSS regions as custom background.

### 4.4 Transcriptome analysis

Marmoset gene IDs were first mapped to human orthologues, and count data from marmoset preimplantation embryos (E‐MTAB7078, 162 samples; E‐MTAB‐9367, 60 samples) were loaded into Seurat (Hao et al., 2024). Human data for zygote to compacted morula stages (GSE36552, 81 samples), blastocysts (hypoblast, epiblast, and trophectoderm; GSE66507, 30 samples), and eight‐cell and compacted morula cells (E‐MTAB‐3929, 112 samples) were loaded into Seurat and merged with the marmoset data. Human sample annotations were updated according to the suggested reannotations of Stirparo et al. (2018).

For differential expression, raw counts from individual cells from the same tissue type within a specific embryo were summed to create a pseudobulk count, that is, different embryos were used as replicates. Each tissue had at least two independent samples. Differential expression was calculated between marmoset and human based on these pseudobulk values, with calculations done using Seurat with the FindMarkers and DESeq2. Differential expression was done for the list of genes relating to KEGG pathway 00603, and *p* values were adjusted inbuilt R function p.adjust with Benjamani‐Hochberg corrections.

For visualization, count data was subsequently normalized to CP10K +1 within Seurat. Some genes were visualized using the violin plot function (VlnPlot). Gene expression was averaged over cell‐type annotations using the AvExpression function, that is, to create a pseudobulk expression for each tissue type and expression of key genes visualized using pheatmap. Expression of genes relating to KEGG pathway 00603 was visualized using pheatmap. Note that pheatmap was also used to cluster genes based on these pseudobulk expression levels. Differential expression between marmosets and humans for each gene and stage/tissue was also indicated in the pheatmap plots.

The relationship between gene expression and methylation levels was visualized using ggplot. We first stratified genes into highly methylated (>95%) and lowly methylated (<5%) genes and plotted expression levels for various tissues for the highly and lowly methylated genes as boxplots.

## AUTHOR CONTRIBUTIONS

TH designed the study; TH and MD wrote the manuscript; LB performed the wet experiments; MD and TM analyzed the RRBS data sets; CD and RB provided the marmoset sperm samples and testicular histology; CAP and TEB performed transcriptome analysis. All authors reviewed and approved the final manuscript.

## FUNDING INFORMATION

This study was supported by the German Research Foundation (grant no. HA1374/19‐1 to TH). TEB and CAP were supported by the Wellcome Trust (WT G117980).

## CONFLICT OF INTEREST STATEMENT

The authors have declared that no conflict of interest exists.

## Supporting information


Figures S1‐S4.

Tables S1‐S4.

## Data Availability

The marmoset RRBS data set has been submitted to the NCBI Sequence Read Archive.
