# Supplementary material for: Age‐related and species‐specific methylation changes in the protein‐coding marmoset sperm epigenome
Source: Aging Cell. 2024 May 16;23(8):e14200. doi: 10.1111/acel.14200 (PMC11320356; doi:10.1111/acel.14200)
Supplement: Supplementary file 1 — Figures S1‐S4. [file ACEL-23-e14200-s002.docx]

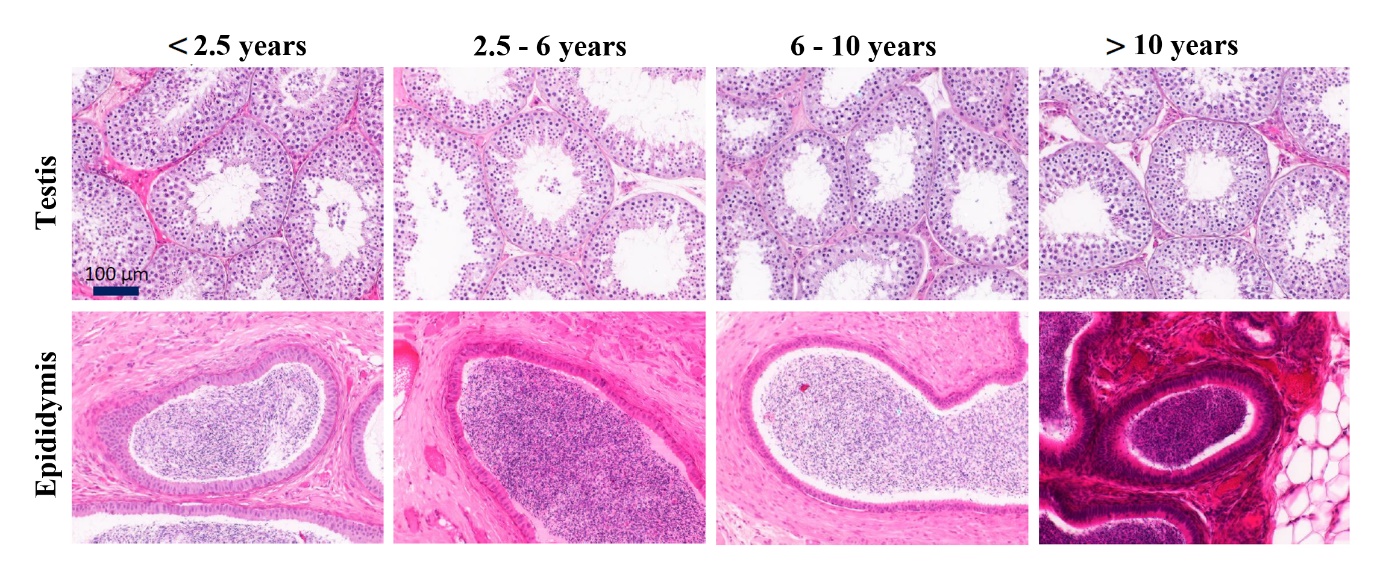


**Figure S1.** Representative histological images of testes (upper row) and epididymides (lower row) of marmosets from the different age groups (n = 4 per group). The tissues were fixed in Bouin’s solution, paraffin-embedded, sectioned at 5 µm, dewaxed and finally hematoxylin/eosin-stained according to standard procedures. All testes show complete spermatogenesis. In the testes, a tubule with almost mature spermatids shortly before spermatogenesis (stage II or III) can always be seen in the center of the image. The epididymides of all age groups are filled with spermatozoa, indicating ongoing spermatogenesis. The scale bar corresponds to 100 µm in all photomicrographs.


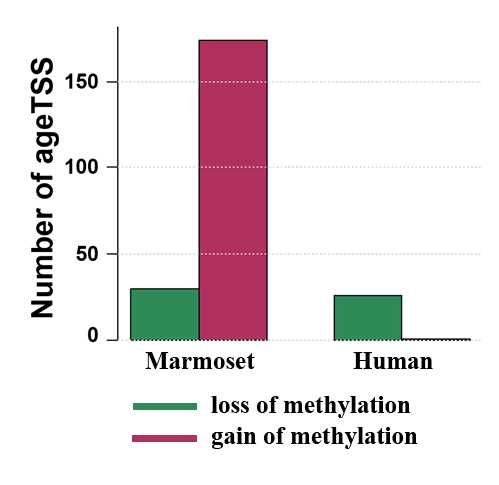


**Figure S2.** Genes with ageTSS in marmoset and human sperm. The bar charts represent the number of significantly (adjusted *p* < 0.05) differentially methylated TSS regions in marmosets and humans, respectively. In marmosets most ageTSS show an increase of methylation with age (mauve bars), whereas in humans almost all genes loose methylation with age (green bars).


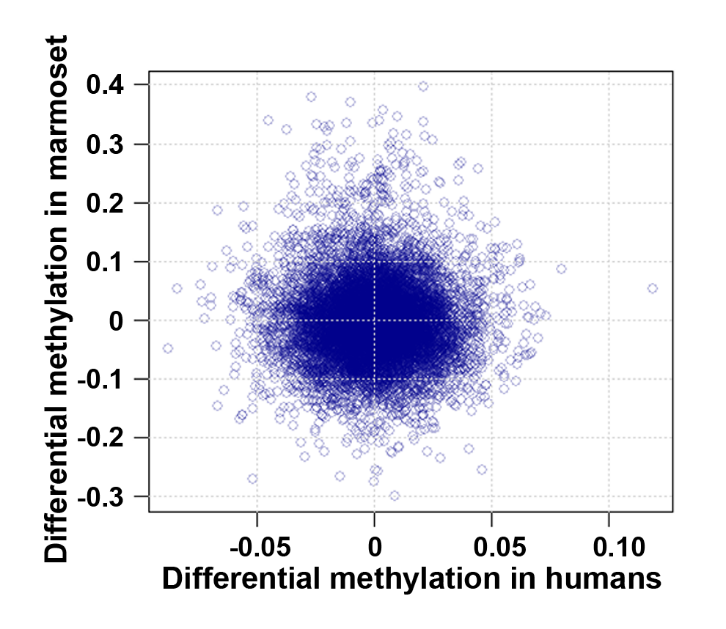


**Figure S3.** Correlation of regression coefficients of age. Scatter plot of age-dependent differential methylation values (on the M-value scale) between humans and marmosets. Only a very week correlation (Pearson's product-moment correlation -0.021) of the age effects can be observed between the species.


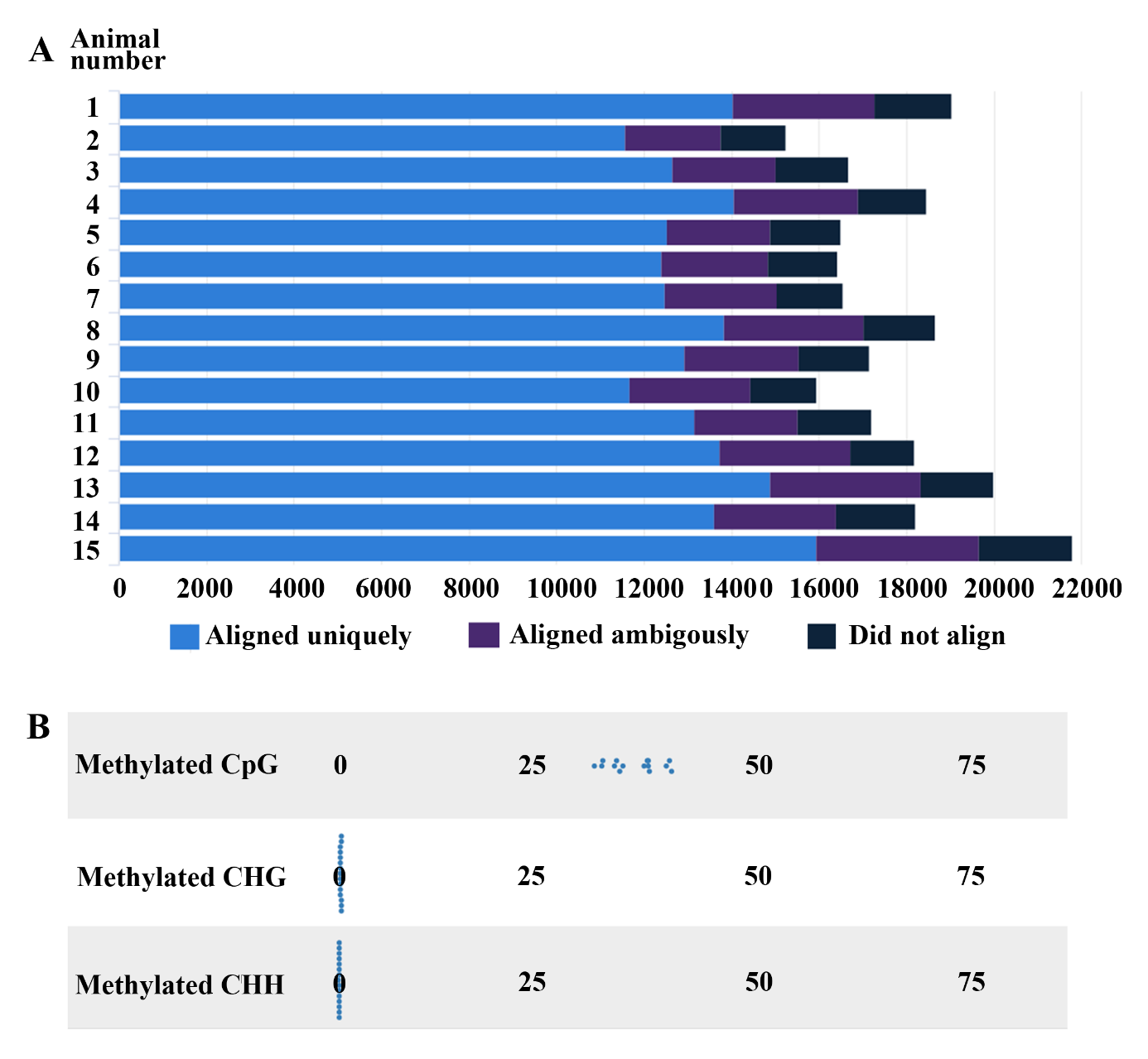


**Figure S4.** QC metrics based on the Bismark alignment and methylation call report as summarized by MultiOC. (**A)** Bismark alignment rates in number of reads for libraries 1-15. (B) Overall cytosine methylation. For each library the percentage methylation of cytosines in CpG, CHG, or CHH context (H can be either A, T or C) is marked as a dot. There is genome-wide methylation in the CpG context (upper panel), but almost none of non-CpG cytosines (lower panels).
